# Immunosuppression Masking a Urological Emergency: A Case Report of Bilateral Infected Obstructed Kidneys

**DOI:** 10.7759/cureus.97923

**Published:** 2025-11-27

**Authors:** Philip Abolanle, Maike Eylert

**Affiliations:** 1 Urology Department, Royal Gwent Hospital, Newport, GBR

**Keywords:** immunosuppression, infected obstructed kidney, organ transplant, sepsis, ureteric stones

## Abstract

An infected obstructed kidney is a urological emergency with the potential to cause life-threatening sepsis. It is often due to ureteric calculi and requires urgent decompression by ureteric stent or percutaneous nephrostomy. We present a patient who presented with bilateral obstructed kidneys secondary to ureteric stones while on immunosuppressive therapy.

A middle‑aged woman with prior bilateral lung transplants on tacrolimus and prednisolone presented with acute abdominal pain, fever, tachycardia, and hypotension. Clinical examination was unremarkable, and blood tests showed normal inflammatory markers and renal function. CT imaging revealed a 6 mm right distal ureteric calculus and a 4 mm left vesicoureteric junction calculus with subtle obstructive features. Despite atypical findings, urological intervention was pursued, given ongoing sepsis and absence of alternative pathology.

The patient underwent bilateral ureteric stenting and was admitted to intensive care, recovered well, and was discharged. Elective ureteroscopy and laser lithotripsy achieved stone clearance six weeks later, with no recurrence at six‑month follow‑up. She has been discharged from urology follow-up. The case underscores the challenges in diagnosis and management of immunosuppressed patients, where atypical clinical presentations may cause diagnostic uncertainties. Multidisciplinary coordination is essential to achieve optimal outcomes in such complex scenarios.

## Introduction

Sepsis is a life-threatening organ dysfunction due to a dysregulated host response to infection and is considered a major cause of morbidity and mortality. The UK Sepsis Trust reports an estimated annual mortality related to sepsis of 48,000 [1,2]. The mainstay of sepsis management continues to emphasize early identification, prompt diagnostic evaluation, timely initiation of antimicrobial therapy, and rapid haemodynamic resuscitation [2]. The conceptual framework has recently shifted from viewing it predominantly as a systemic inflammatory response to recognizing it as a dysregulated host immune reaction to infection, ultimately resulting in organ dysfunction [3].

Immunosuppression may result from either congenital or acquired deficiencies in immune function, such as those arising from critical illness or iatrogenic interventions, which significantly elevate the risk of infection and sepsis [3,4]. Immunosuppression can alter the body's response to sepsis, leading to often varying and confounding clinical presentations that may create diagnostic uncertainty and delay in intervention. Understanding the peculiar challenges posed by this interplay of factors is essential for optimal outcome [4].

The infected kidney obstructed (infected hydronephrosis), a urological emergency with the potential to progress to urosepsis, is defined as a bacterial infection in a hydronephrotic kidney [5]. The standard management of infected hydronephrosis secondary to ureteric calculi involves prompt decompression of the renal collecting system. Percutaneous nephrostomy offers the benefits of avoiding general anesthesia and minimizing instrumentation of the urinary tract, and is typically performed by an interventional radiologist or a trained urologist. Alternatively, retrograde ureteric stenting under general anesthesia provides internal drainage, may facilitate earlier patient discharge, and offers improved access for subsequent ureteroscopy, if required.

Timely recognition of sepsis and the prompt initiation of targeted, aggressive therapy remain the cornerstone of effective management. Optimal treatment entails early fluid resuscitation aimed at restoring tissue perfusion, alongside the rapid administration of appropriate antimicrobial agents [3]. The National Institute for Health and Care Excellence recommends the implementation of the “Sepsis Six” bundle within the first hour following recognition of sepsis. This involves administration of oxygen if required, blood tests, microbiology sampling, and intravenous administration of broad-spectrum antibiotics at maximum recommended dosage, among other measures [6]. Sepsis has been found to trigger a complex immunological cascade characterized by persistent inflammation alongside profound immunosuppression. The immunosuppression is now increasingly recognized as a critical contributor to sepsis-related mortality and stems from a disruption of immune homeostasis, accelerated apoptosis of immune effector cells, expansion of immunosuppressive cell populations, heightened release of anti-inflammatory cytokines, and upregulation of immune checkpoint pathways [7]. 

## Case presentation

A middle-aged lady was referred to the surgical assessment unit with a sudden onset of upper abdominal pain, nausea, vomiting, malaise, and fevers. She had a fever as high as 40°C, was tachycardic with a heart rate of 110 bpm, and hypotensive with a blood pressure of 80/50 mmHg on admission. She had an oxygen saturation of 97% on room air and a respiratory rate of 18 cycles per minute. She had no history of previous similar pain and no history of gallstones or kidney stones. She had a medical background of interstitial lung disease, for which she had bilateral lung transplants just nine months earlier, polymyositis, and left ventricular systolic dysfunction. She was on maintenance tacrolimus and prednisolone for immunosuppression following the lung transplant. 

On initial clinical evaluation, the chest was clinically clear, and the abdomen was diffusely tender to palpation with no specific signs of peritonitis. Resuscitation was promptly commenced within the first hour of admission, with 1 L of intravenous fluids (Hartmann's solution) and antibiotics (amoxycillin, gentamicin, and metronidazole as per hospital protocol), and blood samples were taken for laboratory analysis. Urine and blood cultures were also sent. A venous blood gas showed a lactate of 6 mmol/L with other parameters within normal limits. Results of full blood counts, urea and electrolytes, liver function tests, and C-reactive protein were barely abnormal (Tables 1, 2).

**Table 1:** Full blood count results showing relatively normal values.

Parameter	Result	Reference value
White cell count	3.3	4.0-11.0 x 10^9^/L
Haemoglobin	146	115-165 g/L
Platelets	193	150-400 x 10^9^/L
Neutrophils	3	1.7-7.5 x 10^9^/L

**Table 2:** Results showing values for kidney function test, C-reactive protein, and lactate. Note the markedly raised lactate despite normal inflammatory markers.

Parameter	Result	Reference value
Sodium	144	133-146 mmol/L
Potassium	3.1	3.5-5.3 mmol/L
Urea	6.6	2.5-7.8 mmol/L
Creatinine	87	46-92 umol/L
C-reactive protein	<10	<10 mg/L
Lactate	6	0.5-2.2 mmol/L

**Figure 1:**
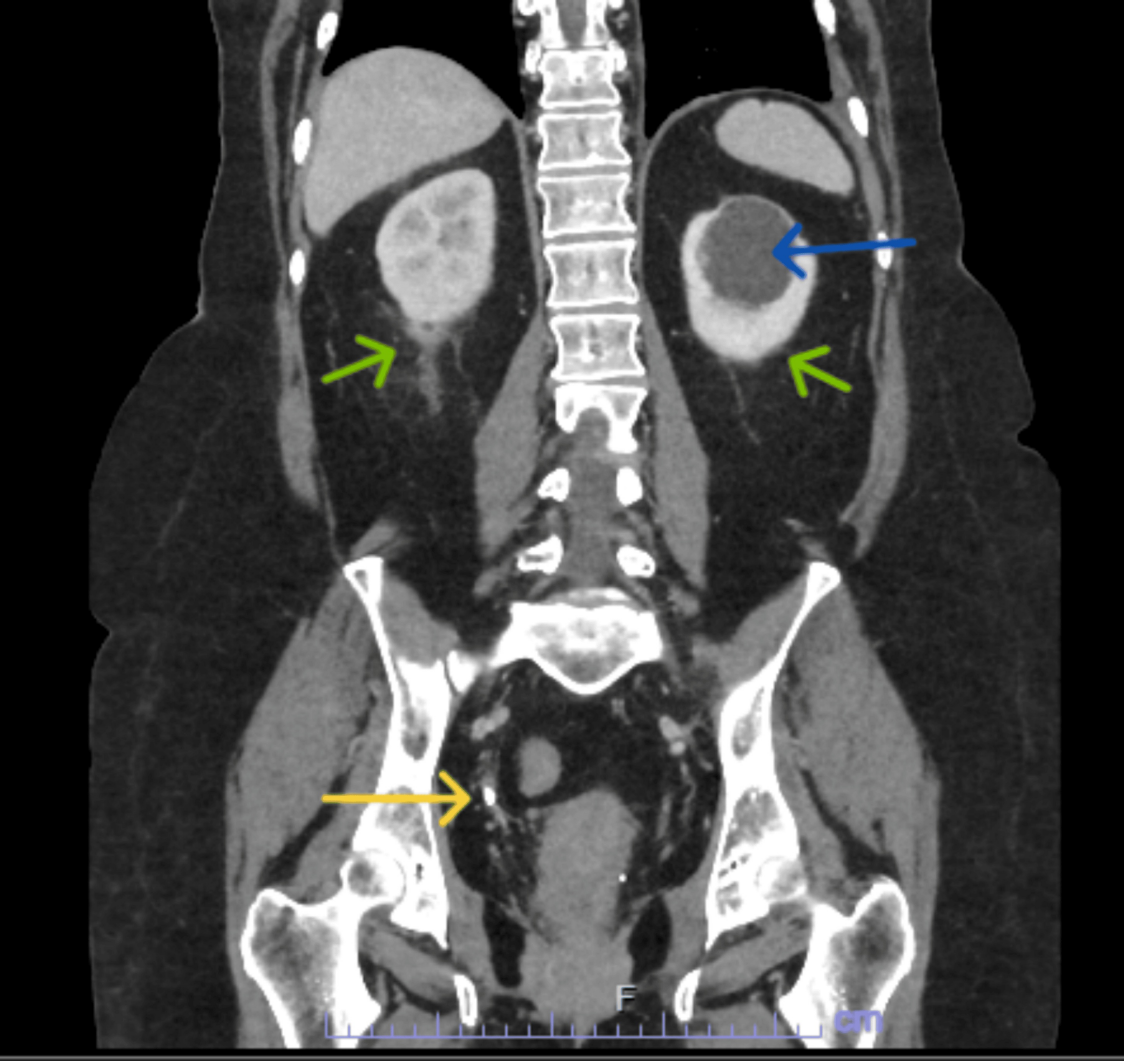
Coronal view of CT abdomen and pelvis showing a distal right 6 mm ureteric stone (yellow arrow). Also note bilateral perinephric stranding (green arrows) and a parapelvic cyst on the left (blue arrow).

**Figure 2:**
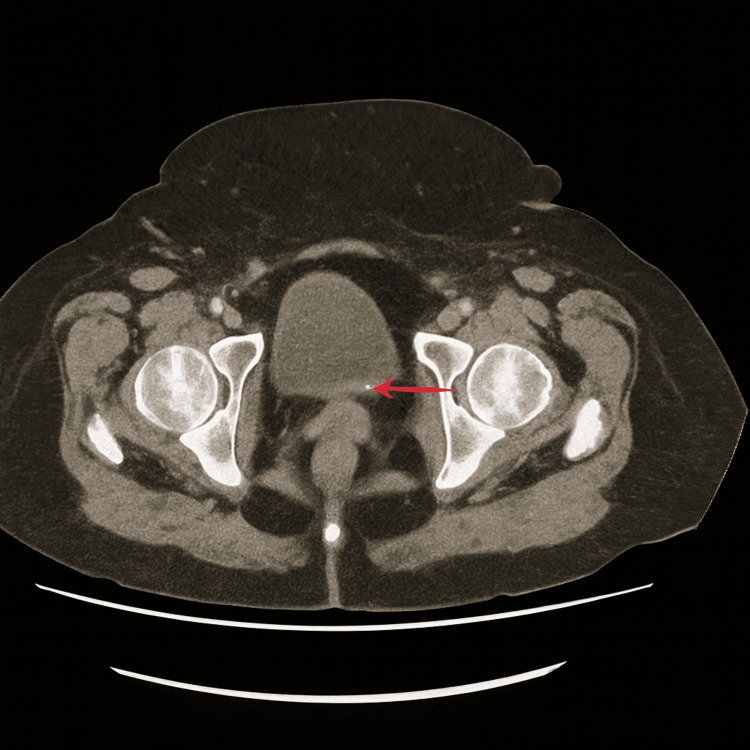
Axial view of CT abdomen and pelvis showing a stone at the left vesicoureteric junction (red arrow).

A plain chest radiograph showed no obvious abnormality. A decision was made to perform a CT scan of the abdomen and pelvis to investigate the source of sepsis. This showed a 6 mm calculus in the lower right ureter (Figure 1) and a 4 mm calculus at the left vesicoureteric junction (Figure 2). There was mild dilatation of the collecting systems bilaterally and a further 5 mm calculus in the lower pole of the left kidney.

Initially, it was unclear whether the ureteric stones were responsible for the patient's clinical condition. The presentation, characterized by upper abdominal pain in the absence of urinary symptoms, flank pain, or renal angle tenderness, was atypical. This uncertainty was compounded by imaging findings of only mild hydronephrosis and perinephric stranding, alongside relatively stable renal function and inflammatory markers. The respiratory medicine team was consulted, and following discussion with the lung transplant team, it was advised that immunosuppressive therapy be continued due to the significant risk of graft rejection in this case. Despite administration of a further 2 L of intravenous fluids over the next two hours, the patient's blood pressure continued to decline, reaching 70/40 mmHg, accompanied by clinical deterioration. Given the worsening condition, urgent intervention was deemed necessary, and the patient was promptly scheduled for emergency surgery to address the ureteric calculi. Our preferred method of management for obstructing calculi is ureteric stenting, as it provides rapid decompression and allows for easier access to the ureter for definitive stone management. In this case, the minimally dilated pelvicalyceal system would​​​​​ have made a nephrostomy insertion technically difficult and likely unsuccessful.

The patient was commenced on vasopressors and hydrocortisone while being wheeled into theatre due to septic shock, and had bilateral ureteric stents inserted under general anaesthesia. Retrograde pyelogram intraoperatively showed mildly dilated collecting system bilaterally, and drainage of purulent urine was observed from both stents. She was then admitted directly into the intensive care unit, where she continued to receive care with vasopressor support for 24 hours until her blood pressure stabilised around 130/70 mmHg. Her lactate levels showed improvement postoperatively and normalized within 48 hours. She was stepped down to the ward after 72 hours in intensive care. 

Over the next few days, she became clinically better, although her inflammatory markers transiently became elevated. Her blood and urine cultures returned positive for *Escherichia coli* and *Proteus mirabilis,* respectively, which were treated with appropriate antibiotics based on sensitivity patterns. Her hospital stay was complicated by *Clostridium difficile* and herpes simplex virus infection, which developed about five days into admission, and these were managed with microbiological guidance. Remarkably, she did not suffer any respiratory dysfunction or infection during the admission. She was discharged safely after two weeks and had ureteroscopy and laser fragmentation of stones six weeks later. An interval CT scan six months later showed no new formation of stones. She has been discharged from urology follow-up. 

## Discussion

Sepsis remains a leading cause of morbidity, mortality, and healthcare expenditure worldwide [1]. It is estimated that approximately 25% of sepsis cases originate from infections within the urinary tract. However, the mortality associated with urosepsis appears to be lower compared to sepsis arising from intra-abdominal or other sources. The infected obstructed kidney is a urological emergency that, often caused by a ureteric calculus, requires urgent decompression of the collecting system. This is usually achieved either by insertion of a ureteric stent or a percutaneous nephrostomy [5]. 

There is an expanding cohort of solid organ transplant (SOT) recipients globally. This is driven by refinements in general medical care and surgical techniques, and advancements in immunosuppressant regimens resulting in better survival rates [8]. Our patient in this report had received a bilateral lung transplant nine months earlier and made an excellent recovery with a return to her normal daily activities and a good quality of life. This reflects the advances in the field of solid organ transplantation, resulting in improved patient outcomes. She had also been on maintenance immunosuppressants and had been able to manage with a good quality of life. The underlying condition that resulted in organ failure, combined with perioperative and postoperative stress, as well as the long-term immunosuppressant drug treatments, predispose transplant recipients to an increased incidence and spectrum of infections [8,9]. In some patients, infections can progress to severe sepsis and septic shock. Sepsis remains a leading contributor to mortality among recipients of organ transplants [8]. Our index patient had bilaterally infected, obstructed kidneys, which is well known to cause urosepsis. The rapid onset of her symptoms and the quick progression to shock represent the need for quick sepsis identification in this patient group, as they can be more vulnerable to infections due to impaired immune function.

The prevailing definition of sepsis may also be suboptimal for SOT recipients, as they often exhibit attenuated clinical manifestations. They may have normal temperatures and unremarkable white cell counts [8,10]. Our patient presented with fever, but the inflammatory markers (white cell count and C-reactive protein) were normal. It has been shown that SOT recipients often present with blunted symptoms and less pronounced clinical and radiologic findings [11]. This is to be expected, as immunosuppression can blunt the inflammatory response and decrease the signs and symptoms of infection, whether in the acute or chronic setting. Our patient presented with upper abdominal pain atypical of ureteric colic, which usually manifests as loin to groin pain. The abdominal examination findings were non-specific, with no flank tenderness demonstrated. This clinical presentation produced clinical uncertainty regarding the CT scan findings. Our experience underscores the need for healthcare professionals involved in the care of SOT patients to have a high index of suspicion for the presence of sepsis. In some instances, tailored definitions can help detect sepsis much quicker. de Carvalho et al. advised that patients with prior or chronic allograft dysfunction who present with a decline in baseline graft function, when accompanied by a suspected or confirmed infection, may be appropriately diagnosed with severe sepsis [12]. 

The optimal management strategy for immunosuppressive therapies during episodes of severe sepsis remains undefined by current consensus. Notably, the rapid discontinuation or aggressive withdrawal of glucocorticoids must be avoided, given the potential for precipitating symptoms of adrenal insufficiency. In cases of septic shock, especially among patients exhibiting inadequate response to fluid resuscitation and vasopressor support, hydrocortisone administration should be considered as part of the treatment approach [8]. Our index patient continued to receive immunosuppressive treatment despite being septic due to the high risk of graft rejection, as advised by the transplant team. She also had hydrocortisone administered as part of treatment for septic shock.

The treatment for acute ureteric obstruction with sepsis is urgent decompression of the obstructed kidney. This is usually achieved either with the insertion of a ureteric stent or percutaneous nephrostomy. Studies have suggested both methods have high success rates and no significant differences in patient outcomes, but with differing outcomes in patients' quality of life [5,13-16]. The decision on which option of care is offered is usually guided by local policies and the availability of resources. A ureteric stent may also be preferred in ureteric obstruction due to calculi, as it facilitates access to the ureter for subsequent ureteroscopic treatment. In our centre, insertion of a ureteric stent is the preferred modality of intervention due to the above factors and the limited availability of interventional radiologists out of hours. Ramsey et al. recommended the implementation of a "stent first where possible" strategy to help decrease the need for out-of-hours nephrostomy in patients with infected hydronephrosis, acknowledging that nephrostomy may still be necessary for the most critically ill cases [5]. 

## Conclusions

This case underscores the diagnostic challenges in immunosuppressed patients, where atypical presentations of ureteric stones and urosepsis may cause uncertainty. Despite the absence of classical features such as flank pain, renal angle tenderness, and elevated inflammatory markers, bilateral ureteric calculi with mild hydronephrosis were the underlying cause of sepsis. Clinicians should maintain a high index of suspicion for urosepsis in immunocompromised individuals, even when typical clinical and radiological signs are lacking. Multidisciplinary coordination remains essential to achieve optimal outcomes in such complex scenarios.
